# The Prediction of Enterprise Stock Change Trend by Deep Neural Network Model

**DOI:** 10.1155/2022/9193055

**Published:** 2022-08-02

**Authors:** Guifen Ma, Ping Chen, Zhaoshan Liu, Jia Liu

**Affiliations:** ^1^Accounting Institute, Guangzhou Huashang College, Guangzhou 511300, Guangdong, China; ^2^Graduate School, Nueva Ecija University of Science and Technology, Cabanatuan 3100, Philippines; ^3^School of Economics and Management, Taishan University, Taian 271000, Shandong, China; ^4^International College, Krirk University, Maha Nakhon, Bangkok 10220, Thailand

## Abstract

This study aims to accurately predict the changing trend of stocks in stock trading so that company investors can obtain higher returns. In building a financial forecasting model, historical data and learned parameters are used to predict future stock prices. Firstly, the relevant theories of stock forecasting are discussed, and problems in stock forecasting are raised. Secondly, the inadequacies of deep neural network (DNN) models are discussed. A prediction trend model of enterprise stock is established based on long short-term memory (LSTM). The uniqueness and innovation lie in using the stock returns of Bank of China securities in 2022 as the training data set. LSTM prediction models are used to perform error analysis on company data training. The 20-day change trend of the company's stock returns under different models is predicted and analyzed. The results show that as the number of iterations increases, the loss rate of the LSTM training curve keeps decreasing until 0. The average return price of the LSTM prediction model is 14.01. This figure is closest to the average real return price of 13.89. Through the forecast trend analysis under different models, LSTM predicts that the stock change trend of the enterprise model is closest to the changing trend of the actual earnings price. The prediction accuracy is better than other prediction models. In addition, this study explores the characteristics of high noise and complexity of corporate stock time series, designs a DNN prediction model, and verifies the feasibility of the LSTM model to predict corporate stock changes with high accuracy.

## 1. Introduction

In recent years, scholars have been exploring the stock market. They were constantly developing new technologies, new models, and better methods [1, 2]. Such continuous investment and the emergence of many research results make stock market research increasingly mature. As the stock market has grown, time series analysis has brought a corresponding rise. The idea of time series is to use the relationship between variables and time in the series to establish a statistical forecasting model [3]. On the basis of the time series method, time series models such as autoregression, moving average, autoregressive moving average, generalized autoregressive conditional heteroscedasticity, and Monte Carlo simulation are generated [4]. However, financial time series objects are affected by many factors, such as stock data. It tends to be nonstationary, nonlinear, and noisy. The financial time series object is different from the previous time series method, which only involves unilateral influencing factors of time. Therefore, the traditional time series method is unsuitable for stock data, and the prediction performance is not ideal [5–7]. With the breakthrough of the Internet and computer technology, the development of data storage technology has ushered in the era of big data. People are constantly exposed to a large amount of data and information from computers and mobile phones for huge amounts of data. Obtaining useful and human-wanted information from it has become the focus of research [8]. Large amounts of data are generated every day due to the timeliness of the stock market. The stock market has accumulated enough historical data. Experts and scholars have obtained a large amount of research data and can research the stock market [9]. The price of a stock is affected by several factors. This makes forecasting stock prices not so easy. However, with the development of machine learning technology, it is possible to dig out the information that is extremely important for stock forecasting from the massive information. Therefore, the work of stock forecasting is still of great value and significance [10].

Jalali and Heidari established a stock market forecast model based on grey system theory and made specific forecasts for stock prices [11]. With the increasing improvement of neural network learning theory, scholars have carried out mathematical modeling and prediction on the stock market. Peng et al. used a wavelet neural network to predict the trend of stocks [12]. Nti et al. used echo vector machines to predict the trend of stocks [13]. Livieris et al. used recurrent neural networks (RNN) to predict the trend of stocks [14]. Ecer et al. predicted the performance of the Istanbul Stock Exchange based on an artificial neural network (ANN) and regression model. According to the comparison, the prediction effect of the neural network was better than that of other regression models [15]. Lv et al. improved the basic backpropagation neural network (BPNN) algorithm. In the experimental results, the prediction accuracy of the improved BPNN is high, and its convergence speed is higher than that of the standard BPNN [16]. Kumar made short-term forecasts for stock prices. Through the stock price prediction analysis of different models of the radial basis function (RBF) network, BPNN, and Elman network, it is concluded that the predictive ability of the feedforward neural network is lower than that of the feedback neural network [17]. Chung and Shin used a genetic algorithm to improve the performance of technical analysis indicators and optimized parameters in the trading system. The development trend of price changes can be found more easily [18]. Nandy et al. used the random forest (RF) algorithm to establish a stock prediction model, and the input features were input through different technical indicators, which made the prediction accuracy of the results very high [19]. Qiu et al. used an improved LSTM model to predict stock indices. The results show that the prediction accuracy of the improved LSTM model is higher than that of the traditional BPNN and the standard LSTM model. Additionally, the prediction volatility of its model on Vestak stock data is small [20]. Mehtab and Sen used python to build a long short-term memory (LSTM) model. They used the model to analyze and predict historical data on steel transaction prices [21]. Ni et al. predicted stock trends from the perspective of market sentiment and established a word embedding stock prediction model based on deep learning methods. The model can extract effective information from news, analyze stock investors' views on current stock holdings during this period, and predict the trend of the next wave of stocks [22]. In the related research on stock forecasting and analysis, scholars use random forest algorithms to predict the stock trend. However, the prediction accuracy of this algorithm still has a large gap compared with the deep neural network (DNN). Therefore, this study aims to build a trend prediction model for company stock changes based on LSTM.

Firstly, the relevant theories of stock forecasting are discussed. Problems with stock forecasting are raised. Secondly, the inadequacies of DNN models are discussed. An LSTM-based corporate stock forecast trend model is established. The innovation lies in using the stock return price of BOC Securities in 2022 as the training data set and conducting error analysis on enterprise data training through the LSTM prediction model. Under different models, the trend of the company's stock earnings price over 20 days is predicted and analyzed.

## 2. Design of LSTM-Based Corporate Stock Change Trend Model

### 2.1. Stock Forecast Theory

**Figure 1:**
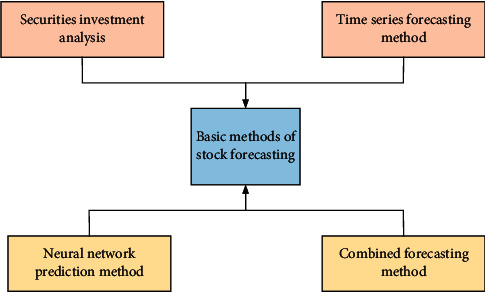
Basic methods of stock forecasting.

In the stock market, the regularity of the development and change of things is found through the precise statistics of movement changes. This law has a great prediction on the trend of future development and change. Changes in technical charts and indicators record past movements in the stock market. Therefore, they carry the laws of market changes. Technical analysis is a common tool for investors to operate stocks. Technical analysis includes two aspects: various graphics of index (price) movement and analysis of trend changes of various technical indicators. Technical analysis is to summarize and count the past trend of the index, extract the direction and forecast of the future operation of the index, and provide investors with operational guidance. The basic method of stock forecasting is shown in Figure 1.

In Figure 1, the securities investment analysis method comprehensively analyzes various information affecting the value or price of securities through various professional analysis methods to judge the value or price of securities and their changes, which is an important link in securities investment. There are mainly basic analysis and technical analysis. The fundamental analysis mainly analyzes the internal factors of the issuing company. The analysis of the internal factors of the issuing company mainly analyzes the internal factors of the issuing company. The analysis of the internal factors of the issuer, such as the issuer's credit rating, financing capability, operation and management status, and long-term planning, provides a basis for investors to choose a reliable investment object. It mainly refers to analyzing the trend of changes in securities prices using various technical methods, which provides a basis for investors to observe and judge the stock market trend and make securities trading decisions. Time series forecasting uses the data of the past period to predict the information of the future period, including continuous forecasting (numerical forecasting, range estimation) and discrete forecasting (event forecasting), with very high commercial value. The neural network prediction method is to predict the stock trend through ANN, use the time series composed of stock transaction data, simulate the functional relationship between input and output data through self-learning, and apply the function to predict future stock prices. The combined forecasting method is forecasting using two or more different forecasting methods. It can be a combination of quantitative or qualitative methods. But in practice, more qualitative and quantitative methods are used. The main purpose of combination is to comprehensively utilize the information provided by various methods to improve the prediction accuracy as much as possible [23]. Commonly used raw stock data are shown in Table 1.

Technical indicators are widely used in actual stock market operations. Commonly used stock technical indicators are as follows: moving average (MA) is a statistical analysis method. The securities prices (index) in a certain period are averaged. The averages of different times are connected to form a MA. A technical indicator is used to observe the trend of securities price changes [24, 25]. The calculation expression is shown as follows:

(1) $$\begin{array}{c} MA_{n}=\left(C_{1}+C_{2}+C_{3}+C_{4}+\cdots +C_{n}\right)÷n. \end{array}$$

*C*_1_,…, *C*_*n*_ are the sum of the closing prices of *n* days, and *n* is the number of days. The deviation rate (*Y* value) is a technical indicator derived from the moving average principle. Its function is mainly to measure the degree of deviation of the stock price from the moving average during the fluctuation process to obtain the possible retracement or rebound caused by the deviation of the stock price from the moving average trend when the stock price fluctuates violently. Stock prices are within the normal range and continue their trend. The calculation of the deviation rate is shown as follows:

(2) $$\begin{array}{c} N_{b}=\left[\left(p-N_{a}\right)÷N_{a}\right]\times 100\%. \end{array}$$

*N*_*a*_ and *N*_*b*_ are the daily multiplication rate and daily moving average price, respectively. *p* is the closing price of the day. Moving average convergence divergence (MACD) is based on the construction principle of the moving average, smoothing the closing price of the stock price and then calculating the arithmetic mean value. It is a trend indicator. The MACD indicator is a double smoothing operation using fast (short term) and slow (long term) moving averages and their signs of convergence and separation. MACD is developed according to the principle of moving average; in addition to the defect that moving average sends false signals, it also retains the effect of moving average. Therefore, the MACD indicator has the characteristics of moving average trend and stability. It is a technical analysis indicator used to judge the timing of buying and selling stocks and predict the rise and fall of stock prices. The MACD consists of the positive and negative difference (DIF) and the difference exponential average (DEA). DIF is calculated as in the following equations:

(3) $$\begin{array}{c} EMA\left(12\right)=\frac{2}{12+1}\times p+11/\left(12+1\right)\times EMA{\left(12\right)}_{T}, \end{array}$$

(4) $$\begin{array}{c} EMA\left(26\right)=\frac{2}{26+1}\times p+\frac{25}{\left(26+1\right)}\times EMA{\left(26\right)}_{T}, \end{array}$$

(5) $$\begin{array}{c} DI\;F=EMA\left(12\right)-EMA\left(26\right). \end{array}$$

EMA(12) and EMA(26) are the fast smooth moving line on the 12th and the slow smoothing average on the 26th, respectively. *p* is the closing price of the day. EMA(12)_*T*_ and EMA(26)_*T*_ are the EMA of the previous day. After the DIF is obtained, the individual DIF can also perform market forecasting. However, for a more reliable signal, DEA is calculated as follows:

(6) $$\begin{array}{c} DE\;A=\frac{2}{9+1}\times DI\;FF+\frac{\left(9-1\right)}{\left(9+1\right)}\times DE\;A_{T}. \end{array}$$

DIFF is today's DIF value, and DEA_*T*_ is the previous day's DEA value. The DIFF line is the difference between the 12-day EMA and the 26-day EMA. The DEA line is the 9-day smoothed moving average of the DIFF line. Sentiment indicators and willingness indicators are both technical indicators that analyze historical stock prices. Among them, the popularity indicator pays more attention to the opening price, thus reflecting the popularity of market trading. The willingness indicator pays attention to the closing price and reflects the degree of market willingness to buy and sell. The two indicators analyze stock price fluctuations from different perspectives to achieve the common purpose of tracking the future trend of stock prices. According to On-Balance Volume (OBV), the four elements of stock market technical analysis are price, volume, time, and space. The OBV indicator is a technical indicator that uses the factor of “quantity” as a breakthrough to discover popular stocks and analyze the movement trend of stock prices. It digitizes and visualizes the relationship between the popularity of the stock market, the trading volume, and the stock price and measures the driving force of the stock market by the change in the stock market trading volume to study and judge the trend of the stock price. Regarding volume research, the OBV energy tide indicator is one of the most important analytical indicators. The Relative Strength Index (RSI) analyzes the intention and strength of the market's buying and selling orders by comparing the average closing gain and the average closing decline to make a future market trend. The calculation of RSI is shown as follows:

(7) $$\begin{array}{c} RSI=\left[\bar{h}÷\left(\bar{h}+\bar{d}\right)\right]\times 100. \end{array}$$

**Figure 2:**
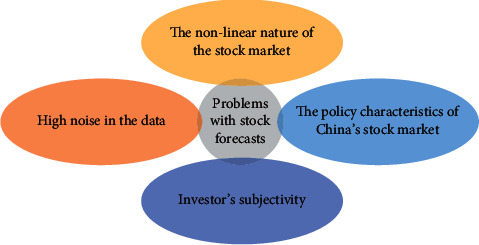
Problems with stock forecasting.

$\bar{h}$ represents the rising average and $\bar{d}$ represents the falling average. The rising average is the average of the rises over a certain day, and the falling average is the average of the falls over the same day. The extent to which a stock price changes outside its normal range is measured. The indicator considers not only the closing price but also recent highs and lows. This avoids the weakness of considering only the closing price and ignoring the true volatility range. It is mainly a technical tool that uses the real volatility of price fluctuations to reflect the strength of price trends and overbought and oversold phenomena and to buy and sell signals before prices have risen or fallen. It mainly studies the relationship between the highest price, the lowest price, and the closing price in the design process. Additionally, it also combines some advantages of the momentum concept, strength indicator, and moving average to judge the market quickly and intuitively. The psychological line, also known as the Majority Rule (MJR), is a popular index based on the research on the psychological trend of investors for buying and selling stocks. The problem with stock forecasting is shown in Figure 2.

### 2.2. Theoretical Basis of the DNN Model

**Figure 3:**
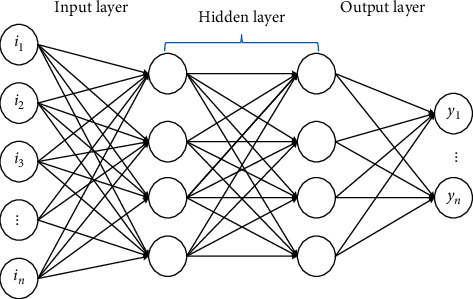
DNN model.

In recent years, some people have begun to apply neural networks to the financial field, hoping to achieve results beyond traditional machine learning methods. The effect of the firm's strategy on various institutions did not disappoint. With reasonable marking and massive factor construction, the neural network-based strategy achieved good results. Neural networks are based on perceptron extensions, and DNN can be understood as neural networks with many hidden layers. The DNN model is shown in Figure 3.

**Figure 4:**
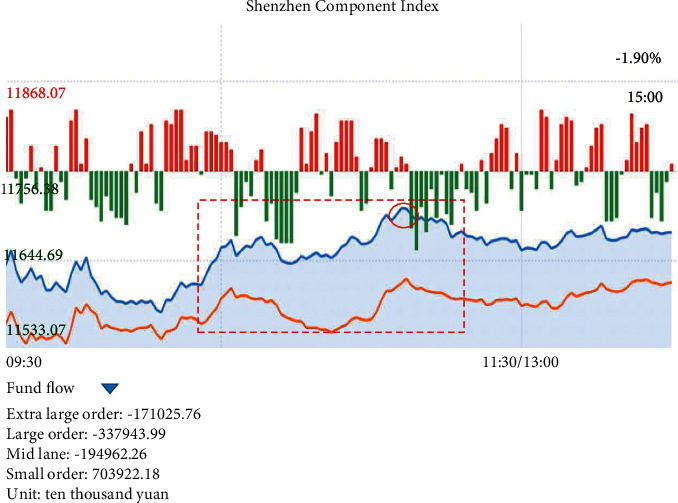
Changes in Shenzhen Component Index stocks (the data comes from 2A01 stocks on April 13, 2022, on CITIC Securities.com).

In Figure 3, *i*_*n*_ is the input of the neuron, and *y*_*n*_ is the output value. However, as the number of hidden layers of the neural network deepens, the optimization function easily falls into the optimal local solution, the performance is not as good as that of the shallower network, and the phenomenon of “gradient disappearance” is more serious. RNN is designed to deal with the problem that ordinary neural networks cannot handle fixed-length sequences. In the RNN, the data is regarded as a sequence of different sections entering the network. The data of each section is combined with the data output from the previous section for input. However, if the length of the time series can be controlled, it does not need to rely on the input mode of the series data of different sections, but the data of a whole time series is transmitted to the network in one go [26]. The changes in Shenzhen Component Index stocks on April 13, 2022, are used as an example for analysis. The changes in Shenzhen Component Index stocks on April 13, 2022, are shown in Figure 4.

**Figure 5:**
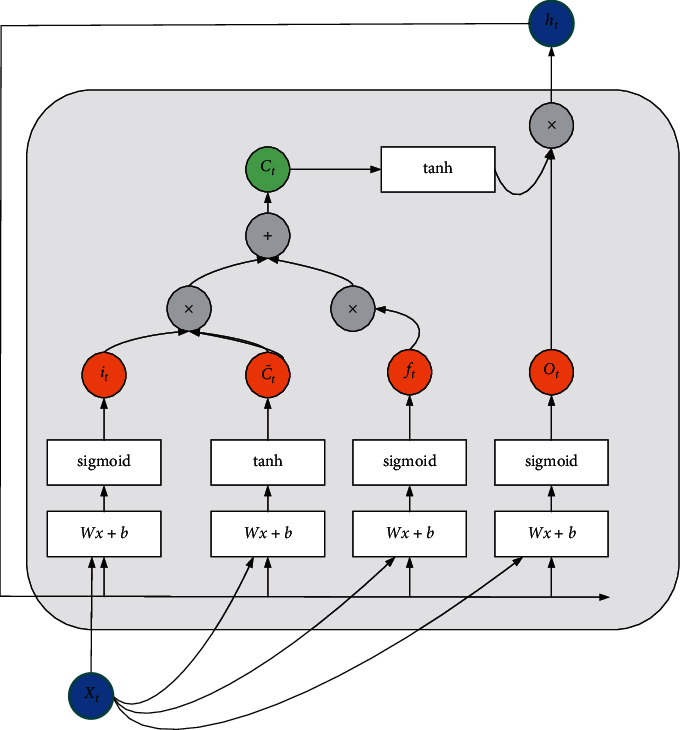
Cell structure of LSTM.

In Figure 4, if the circle position in the figure can be predicted, the data used is in the red box. In the analysis process, the overall situation of this range trend is directly obtained or observed from left to right. However, in the decision-making process, traders basically observe the recent trend of a small segment and then judge based on the recent trend of a large segment. A left-to-right pattern of data input may also not be necessary while constructing a neural network. LSTM is a very powerful time series-based model. They can predict any step backward. An LSTM cell uses five important parameters to model long-term and short-term data. The LSTM cell structure is shown in Figure 5.

In Figure 5, *C*_*t*_ is the unit state, which represents the short-term and long-term memory stored by the unit; *h*_*t*_ is the hidden state, which is the output state information calculated based on the current input, the previous hidden state, and the current unit input to predict the future stock price. In addition, the hidden state also determines whether to use only the memory in the cell state for the next prediction; *i*_*t*_ is the input gate, the information flowing from the input gate into the cell state; *f*_*t*_ is the forget gate, from the current input and the previous cell state, information flowing to the current cell state; *O*_*t*_ is the output gate, information flowing from the current cell state to the hidden state. *X*_*t*_ is the input at the current moment, ${\tilde{C}}_{t}$ is the new candidate value vector created by the tanh layer, *Wx*+*b* is the weighted value of the input vector, and *b* is the bias [27], as shown in the following equations:

(8) $$\begin{array}{c} C_{t}=f_{t}*c_{t-1}+i_{t}*{\tilde{C}}_{t}, \end{array}$$

(9) $$\begin{array}{c} O_{t}=\sigma \left(x_{t}W_{ox}+h_{t-1}W_{oh}+b_{o}\right), \end{array}$$

(10) $$\begin{array}{c} h_{t}=O_{t}*\;\tanh\left(C_{t}\right). \end{array}$$

**Figure 6:**
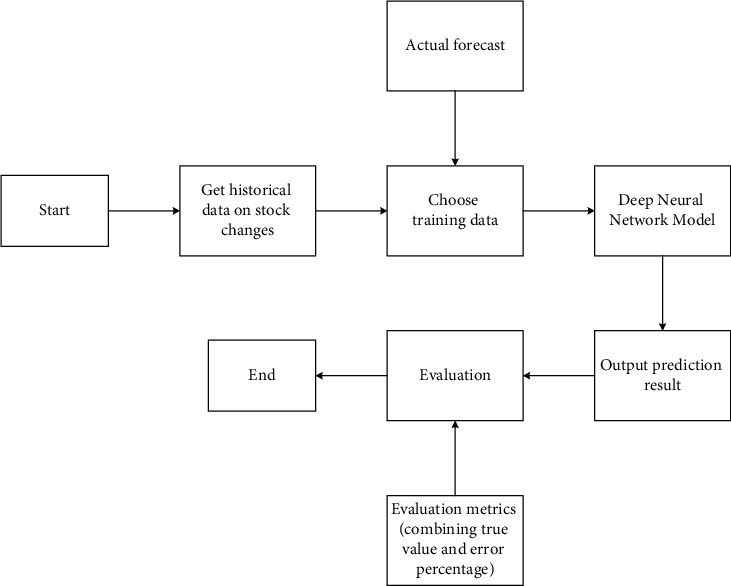
Prediction process of corporate stock change based on the LSTM model.

*C*_*t*−1_ is the cell state at the previous moment, *σ* is the sigmoid layer, and the output result is 0 or 1. 0 means that no information is allowed to pass through. 1 means let all information through. *W*_*ox*_ and *W*_*oh*_ are the connection weight matrix between the output at the current moment and the hidden state of the previous layer, respectively. *b*_*o*_ is the bias. The process of predicting changes in corporate stocks based on the LSTM model is shown in Figure 6.

### 2.3. Model Data Processing

**Figure 7:**
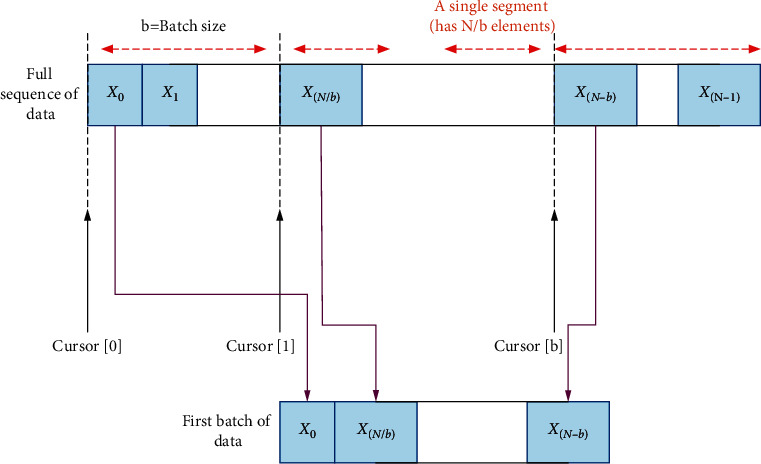
Data generation process.

The data generation process is shown in Figure 7.

In Figure 7, the data set with a total amount of *N* is evenly divided into *N*/*b* sequences, and each sequence contains *b* data points. The divided data of *X*_0_-*X*_*N*−1_ takes the subscript of the element pointed to by the pointer of the current segment as the input. One of the following 1–5 elements is randomly selected as the correct prediction value. The model does not always only predict the data immediately after the current point in time. This can effectively avoid overfitting. The index of the pointer is incremented by one, and the generation of the next data point begins.

## 3. Results Analysis

### 3.1. LSTM Model Error Analysis

**Figure 8:**
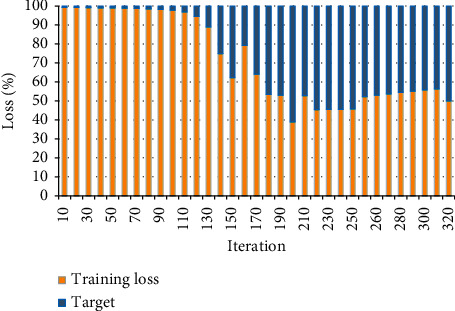
Error curve for training.

According to the experience and foundation of the LSTM model establishment, through multiple prediction analysis experiments on Bank of China Securities, the target accuracy of the loss function is set to 0.005, and the number of iterations is 500. The training error curve is shown in Figure 8.

In Figure 8, as the number of iterations increases, the loss rate of the LSTM training curve continues to decrease until it decreases to 0. At 35 iterations, the LSTM training error drops from 8 to close to 0. The LSTM prediction model meets the training requirements during iterative training, reduces the number of iterations, and saves training time.

### 3.2. Analysis of Training Results of Different Models

**Figure 9:**
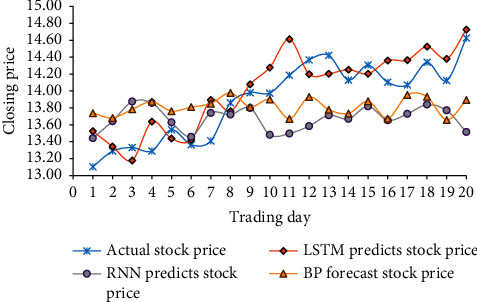
Changes in stock forecasts under different models.

In 2022, BOC Securities stock change data will be used as a data set. The stock data in data set 20 is used as training data through the LSTM model, the RNN model, and the BPNN model. The stock prediction changes under different models are shown in Figure 9.

In Figure 9, the changing trend of the stock return price trained by the LSTM model is most like the changing trend of the actual return price. The actual earnings price in these 20 days averaged 13.89. The average return price of the LSTM prediction model is 14.01, the average return price of the RNN prediction model is 13.60, and the average return price of the BPNN model is 13.78. Therefore, LSTM predicts that the stock change trend of the enterprise model is the closest to the changing trend of the actual income price, and the prediction accuracy is better than other prediction models.

### 3.3. Quantitative Analysis and Discussion of Results

The results show that the loss rate of the LSTM training curve continues to decrease as the number of iterations increases. After 320 iterations, the system achieved 80% of its goal. The model loss rate is reduced to 30%. In addition, the 20-day average stock actual earnings price is 13.89. The data show that LSTM predicts that the stock change trend of the enterprise model is closest to the changing trend of the actual earnings price, and the prediction accuracy is better than other prediction models. Chopra and Sharma [28] researched the fund-stock network prediction model and studied the investment level, distribution, and trend of stock funds according to the importance of stocks. The simulation results show that the investment level of stock funds is better than the average investment level of the market. Equity funds are most inclined to invest in the financial industry and adjust the investment ratio of different industries according to the stock market fluctuation. He et al. [29] evaluated the intrinsic stock value of existing capital flows of state-owned banks. They use the cash flow method to make estimates of free cash flow equity and stock value by looking at regulatory capital. These conclusions have important reference values for estimating the intrinsic value of state-owned bank stocks. Yadav et al. [30] studied the projected shrinkage method of American option pricing. They proposed a finite element plus projection and contraction method for stock price forecasting and verified the efficiency of the proposed method compared with existing methods through numerical simulations. Kumar et al. [31] used the group penalized logistic regression method to study and predict the ups and downs of stock prices. The research screened out 24 important technical indicators and divided them into five different indicator groups. The results show that penalized logistic regression and technical indicators can improve prediction accuracy. Correctly predicting the rising and falling trends of stock prices is an important problem in the financial field. This study builds an LSTM prediction model and uses the stock return price of BOC Securities as a training data set. The simulation results verify the feasibility of the LSTM model for predicting changes in corporate stocks with high accuracy.

## 4. Conclusion

In 2022, through the prediction and analysis of the changing trend of corporate stocks by the DNN model, the stock income price of BOC Securities is used as the training data set. Based on the LSTM prediction model, the enterprise's data training is analyzed for error. Under different models, the stock return price of the enterprise within 20 days can be predicted. The research results show that when the number of iterations is 35, the LSTM training error drops from 8 to close to 0. In 2022, the average actual return price of BOC Securities within 20 days is predicted to be 13.89. The average return price of the LSTM prediction model is 14.01, the average return price of the RNN prediction model is 13.60, and the average return price of the BPNN prediction model is 13.78. The LSTM prediction model meets the training requirements during iterative training, reduces the number of iterations, and saves training time. The changing trend of the stock return price trained by the LSTM model is most like the changing trend of the actual return price. The prediction accuracy is also better than other prediction models. This study studies the characteristics of high noise and complexity of time series of corporate stocks, designs a prediction model of the DNN, and verifies the feasibility of the LSTM model to predict changes in corporate stores with high accuracy. However, there are still some deficiencies to be improved. The main deficiency is that stock changes are not considered due to social factors. This study needs to quantitatively analyze the characteristics of corporate stock data, establish a training model, and further analyze the data to make the prediction results more accurate.

## Figures and Tables

**Table 1:** Commonly used raw stock data.

Commonly used raw data	Illustration
Opening price	The price at which securities are first bought and sold on a stock exchange after the opening of each trading day
Highest price	The highest value of the stock price of the day, generally 110% of the opening
Lowest price	The lowest value of the stock price of the day generally refers to 90% of the opening price
Closing price	The price of the stock after the end of the day's trading
Volume	The number of transactions between buyers and sellers of stocks, which is unilateral
Turnover	The amount of a stock traded on the exchange-traded market during a specific period

## Data Availability

The data that support the findings of this study are available from the corresponding author upon reasonable request.
